# Incidence of sexual dysfunction in men after cardiac surgery in Afshar hospital, Yazd

**Published:** 2011

**Authors:** Seyed Khalil Foruzan-Nia, Mohammad Hassan Abdollahi, Seyed Hossein Hekmatimoghaddam, Seyedeh Mahdiyeh Namayandeh, Mohammad Hadi Mortazavi

**Affiliations:** 1 Department of Cardiac Surgery, Afshar Hospital, Shahid Sadoughi University of Medical Sciences, Yazd, Iran.; 2 Department of Anesthesiology, Afshar Hospital, Shahid Sadoughi University of Medical Sciences, Yazd, Iran.; 3 School of Paramedicine, Shahid Sadoughi University of Medical Sciences, Yazd, Iran.; 4 Yazd Heart Research Center, Afshar Hospital, Shahid Sadoughi University of Medical Sciences, Yazd, Iran.; 5 School of Medicine, Islamic Azad University, Yazd, Iran.

**Keywords:** *Cardiacsurgery*, *Sexualdysfunction*, *Sexuality*, *Libido*, *Impotence*

## Abstract

**Background: **Successful rehabilitation of cardiac surgery patients should include consideration of their sexual activity, but there is paucity of data regarding this matter.

**Objective**: This study determined the incidence and type of sexual dysfunction in our patients.

**Materials and Methods: **Two hundred-seventy nine men with age under 70 years old who had coronary artery bypass graft (CABG), valvular, or other types of cardiac surgery from Dec. 2006 until Dec. 2007 were enrolled in this descriptive-analytical study. They were interviewed before and 12 weeks after the operation in regard to the impact of surgery on their sexuality. The statistical methods used included analysis of variance, Kappa test, and chi-square analysis.

**Results: **The mean age of the patients was 55.7 ± 10.66 (25-69) years. The incidence of sexual dysfunction was 20.1% before, and76.4%, 12 weeks after the operation. P-valueas tested by Kappa test was 0.0001, which means that cardiac surgery had adverse effect on sexual activity of the patients. Types of sexual dysfunction were impotence, premature ejaculation, and decreased or loss of libido in 6.5%, 4.3% and 9.3%, respectively before operation, and 34.8%, 21.5% and 20.1%, respectively 12 weeks after the operation. Concurrence of more than one dysfunction was not reported.

**Conclusion: **Sexual dysfunction is common after cardiac surgery, and sexual counseling is still not being addressed adequately. The role and responsibility of the physician and the rehabilitation nurse becomes evident, together with the need for the patient’s partner to participate in counseling.

## Introduction

The most common cause of death in the world is cardiovascular disease, and the same trend has been predicted to continue until the year 2020 (1).

 Cardiacsurgeries areamong the most stressfuloperations for the patients, and the coronaryartery bypass graft (CABG) is the most common cardiac surgery in the world. Several studies have addressed the relief of symptoms of patient after operation. Successful rehabilitation includes not only the physical and vocational and recreational return, but also the psychosocial return of the patients to their optimal premorbid activity. Studies of the psychosocial outcome of patients after heart surgery have made little reference to sexual activity (2). On the other hand, most of the elderly people remain sexually active, as a global study of sexual attitudes and behaviors showed (3). 

The cardiovascular response to sexual activity has many attributes to the previous stability of the heart (4). Sexual dysfunction may be classified into four main groups: 1/ loss of desire (libido), 2/ erectile dysfunction, 3/ ejaculatory insufficiency, and 4/anorgasmic states (5).

Male sexual dysfunction affects about 10–25% of middle-aged and elderly men (6). Resumption of sexual activity is one of the important factors of psychosocial recovery after cardiac surgery. 

Paucity of data on this subject, especially in Iran, and the importance of sexuality for our patients, as reflected in many of their referrals to clinics, motivated us for this study. Our research aimed at determination of incidence and types of sexual problems after CABG and other heart surgeries, in an effort to obtain data for counseling and rehabilitation of future patients. Many studies have already used a questionnaire for obtaining data about sexual activities (3, 7, 8).

## Materials and methods

In this descriptive-analytical study, 279 male patients under 70 years old who had CABG or other cardiac surgeries in Afshar Hospital, Yazd, from Dec 2006 until Dec 2007 were consecutively (non-randomly) interviewed by a qualified physician in the privacy of office, after an informed consent was signed by them (26 patients were already excluded from the present study because they were not interested). The interviews were conducted before (2-7 days) and 12 weeks after heart surgery using a structured questionnaire (an abbreviated IIEF, the International Index of Erectile Function questionnaire, that was developed and tested by us). Questions were offered verbally, and the physician registered answers of the patients. Each session lasted from 60 to 90 minutes. All patients were married. Variables included the age, cause for operation, method of surgery, and presence of any sexual problem before and after surgery. 

**Statistical analysis**

The statistical methods used in the SPSS-16 software included analysis of variance, kappa test, and chi-square. Any p-value<0.05 was considered as significant.

## Results

Totally 279 patients candidate for cardiac surgery were enrolled in this study. The average age of the cases was 55.7 10.7 years (25-70 years). Two hundred thirty five patients (84.2%) had CABG operation, 33 patients (11.8%) had valvular operation, and 11 patients (4%) were under other cardiac operations. Two hundred twenty one patients (79.2%) were under on-pump procedure, and the rest 58 patients (20.8%) underwent off- pump procedure.

Sexual activity of the cases was asked before operation (in normal status and before the acute illness prompted the surgery). The results are shown in Table I. 

About 79.9% (223 patients) of studied cases had normal sexual activity before the operation, which was similar to this ratio in the society. In 50-59 years old group this ratio was more, and in elder patients it was less. Premature ejaculation was more common in younger cases. In the age group of 50-59 years, impotence and in the age group of 60-69 years loss of libido were significantly more common (p-value= 0.0001). Concurrence of more than one dysfunction was not reported.The sexual problems of the cases before and after operation are shown in table II.

The percentage of patients, who had normal sexual function before operation, was 79.9% which was decreased to 23.7% after operation (6 months later). The incidence of impotence which was 6.5% before operation increase to 34.8% after that; premature ejaculation reached from 4.3% to 21.5%; and loss of libido showed an increase from 9.3% to 20.1%. These variations were evaluated by Kappa test. P-value was 0.0001 (meaningful), which means that cardiac surgery had negative effect on sexual activity of the patients. To better understand what the effects of surgery on these activities are, we divided these variations to 3 groups.

The first group (deteriorated) included the patients who have had normal sexual function before operation but had embroiled to one of the disorders like impotence, premature ejaculation or loss of libido after the surgery.

The second group (without change) consisted of the patients who have had normal sexual activity before and after the operation; or who have had sexual dysfunction before operation and have it unchanged after.

The third group (recovered) included the patients who have had sexual dysfunction before operation but are returned to their normal sexual activity after that (perhaps because of removal of their cardiac problem).This variable was analyzed by multivariate regression test based on age, type of surgery, and the method of surgery (on-pump, off-pump) (Tables III, IV, V).

It is evident that the patients in whom the sexual function is deteriorated by cardiac surgery constituted 60.2% of cases, and the figure is rising with increased age. Sexual function of 35.8% of patients showed no change after operation, especially in younger cases, and fell with increasing age. However, the differences between age groups are not statistically significant (p-value=0.067). Table IV shows changes of the sexual condition in our patients, by type of surgery.

These differences between different types of surgery were analyzed by Fisher Exact test and p-value=0.005 was significant. It means that CABG operation had the most effect in decreasing the sexual activity of patients. Interestingly, improvement in sexual activity has occurred only in some CABG surgery patients. Table V shows changes of the sexual condition in patients, by method of surgery. On-pump procedure was significantly more influential in decreasing sexual activity than off-pump procedure (p-value= 0.0001).

Most of the patients under CABG operation were old, and patients under valve operation were younger (p-value=0.0001). All valve operations were performed with on-pump approach (pvalue= 0.026) and there was no relation between age and on-pump or off-pump procedure (p-value= 0.473). With multinomial logistic regression, it was concluded that by elimination of other variants, risk of deterioration of the sexual activity of patients in CABG operation is 6.57 times more than valve operation (p-value= 0.0005).Also, risk of on-pump procedure in deteriorating the patients' sexual activity condition was 8.27 times more than off-pump procedure (p-value=0.0001). The effect of age was not meaningful in this comparison (p-value=0.688). Multivariate regression analysis was performed for determination of the most important predictors of sexual dysfunction. Older age (OR=1.5, 95% CI= 1.03-2.4), (p=0.036), CABG operation (OR=2.7, 95% CI=1.1-6.6), (p=0.025), and on-pump surgery (OR=9.3, 95% CI= 4.5-19), (p=0.0001) were the significant predictors of sexual dysfunction after open heart surgery.

**Table I:** Sexual activity condition of cases before cardiac surgery in Afshar Hospital according to age, from Dec 2006 to Dec 2007.

** Age (year)**	**25-49**	**50-59**	**60-70**	**Total**
**Sexualactivity**	**No. (%)**	**No. (%)**	**No. (%)**	**No. (%)**
Normal	53 (80.3%)	68 (87.2%)	102 (75.6%)	223 (79.9%)
Impotence	4 (6.1%)	5 (6.4%)	9 (6.7%)	18 (6.5%)
Pre. ejaculation	8 (12.1%)	2 (2.6%	2 (1.5%)	12 (4.3%)
Loss of libido	1 (1.5%)	3 (3.8%)	22 (16.3%)	26 (9.3%)
Total	66 (23.6%)	78 (28%)	135 (48.4%)	279 (100%)

**Table II:** Sexual complaints of cases before and after operation in Afshar Hospital, from Dec 2006 to Dec 2007

** Before**	**Normal**	**Impotence**	**Premature ejaculation**	**Loss of libido**	**Total**
**After**	**No. (%)**	**No. (%)**	**No. (%)**	**No. (%)**	**No. (%)**
Normal	55 (83.3%)	88 (90.7%)	48 (80%)	32 (57.2%)	223 (79.9%)
Impotence	6 (9.1%)	8 (8.3%)	0 (0%)	4 (7.1%)	18 (6.5%)
Pre. ejaculation	2 (3%)	0 (0%)	10 (16.7%)	0 (0%)	12 (4.3%)
Loss of libido	3 (4.6%)	1 (1%)	2 (3.3%)	20 (35.7%)	26 (9.3%)
Total	66 (23.7%)	97 (34.8%)	60 (21.5%)	56 (20.1%)	279 (100%)

**Table III:** Changes of the sexual conditionin patients after cardiac surgery by age, in Afshar Hospital, from Dec 2006 to Dec 2007

** Change**	**Deteriorated**	**Without change**	**Recovered**	**Total**
**Age (years)**	**No. (%)**	**No. (%)**	**No. (%)**	**No. (%)**
25-49	31 (47%)	33 (50%)	2 (3%)	66 (23.6%)
50-59	47 (60.3%)	28 (35.9%)	3 (3.8%)	78 (28%)
60-70	90 (66.7%)	39 (28.9%)	6 (4.4%)	135 (48.4%)
Total	168 (60.2%)	100 (35.8%)	11 (4%)	279 (100%)

**Table IV:** Changes of the sexual condition in patients after cardiac surgery, by type of surgery, in AfsharHospital, from Dec 2006 to Dec 2007.

** Change**	**Deteriorated**	**Without change**	**Recovered**	**Total**
**Surgery**	**No. (%)**	**No. (%)**	**No. (%)**	**No. (%)**
CABG	153 (65.1%)	71 (30.2%)	11 (4.7%)	235 (84.2%)
Valve	10 (30.3%)	23 (69.7%)	0 (0%)	33 (11.8%)
Others	5 (45.5%)	6 (54.5%)	0(0%)	11 (4%)
Total	168 (60.2%)	100 (35.8%)	11 (4%)	279 (100%)

**Table V:** Changes of the sexual condition in patients after cardiac surgery, by method of surgery, in AfsharHospital, from Dec 2006 to Dec 2007

** Change**	**Deteriorated**	**Without change**	**Recovered**	**Total**
**Surgery**	**No. (%)**	**No. (%)**	**No. (%)**	**No. (%)**
On-pump	153 (69.2%)	62 (28.1%)	6 (2.7%)	221 (79.2%)
Off-pump	15 (20.9%)	38 (65.5%)	5 (8.6%)	58 (20.8%)
Total	168 (60.2%)	100 (35.8%)	11 (4%)	279 (100%)

## Discussion

The most common sexual problems which patients with cardiovascular disease are faced with include reduced libido, avoidance of sexual activity, and impotence (9). Resumption of sexual activity after cardiac surgery is a significant factor in returning to usual life. In our study, sexual dysfunction was more prevalent in the older patients before surgery, 35.8% of patients had no change in sexual activity after open heart surgery, 60.2% had deteriorated, and 3.9% had improvement in sexual activity. On the average, cardiac surgery had negative effect on sexual activity of the patients, especially in older cases. The notable limitation of our study was that we did not address all of the different sexual problems (only the 3 most common complaints were assessed), and the detailed questionnaire for international index of erectile function (IIEF) was abbreviated. Also, we did not follow the patients more than 12 weeks to see the long-term changes in their sexual behavior.

In a study on 134 patients to evaluate sexuality after CABG, 92 patients (68.7%) had normal sexual function before operation. In that study, the presence of sexual dysfunction before surgery and delay in getting to a normal work after operation were negative factors effective in resumption of sexual activity after operation. In 39% of patients they encountered reduction in rate of sexual function after operation. About 7% of patients and 35% of their spouses were worried about resumption of normal sexual activity because of occurrence of cardiac phenomena, and 23% of patients expressed their discontent because of symptoms during intercourse (10).

In a study done to estimate the effect of gender on sexual activity change after CABG, it was pointed-out that sexual response is different in men and women. In women there was no considerable decline in rate of sexual activity during the year after operation. Also, in women the fear from cardiac symptoms during intercourse was less than men. Sexual dysfunction in women is chiefly in their sexual desire, but for men is in their sexual arousal (11).

Jenkins *et al* observed 318 patients younger than 70 years who had CABG for 6 months, considering improvement in their psychological, physical activity, occupation, social, familial, gender and distress factors after operation. They found a considerable improvement in comparison to the patients' status before surgery. Also, they noticed that the resumption of normal sexual activity is one of the important aspects of improvement after operation (2).Others have found that after recognition of a cardiac disorder (chest pain, MI, CHF and arrhythmia) or a cardiac interventional method like CABG, angioplasty, pacemaker appliance, heart graft etc., 25% of patients became normal in their sexual function, 25% lost their sexual function completely, and 50% changed to a sexual function less than before (12, 13). Bernardo studied post-cardiac surgery patterns of social and sexual activity, in three periods after operation: 4-10 weeks, 3-6 months, and also 12 months. It was concluded that the most common sign of patients' recovery after cardiac surgery is resumption of their social and sexual activity (14).

In the study done by Heaton in 1996, sexual activity of a group of patients who had been under CABG operation 6-12 months ago was surveyed. About 33% of men had poor erectile function before operation. Eleven out of 30 patients had improvement after operation, and 10 of the patients experienced decline or loss of erectile function after operation. They concluded that CABGhas a significant impact in erectile function (15).

In another study done in 1998 by King about short term recovery from cardiac surgery on 60 men and 60 women, 30 patients of each group were younger than 65 years old. They concluded that sexual function disorders are more common in women than men (16).

In the present study, of the group that was sexually active prior to surgery, 35.8% retained sexual activity. Sexual dissatisfaction prior to surgery played a negative role in resumption of sexual activity, and may be used by CABG patients as an excuse not to resume sexual activity and retreat from marital relation. The higher incidence of fear among those cases who resumed sexual activity, in comparison to those who did not, may be an indication of that feeling being enhanced or developed once the decision is made to resume it. Depression, as a self-reported feeling, and not measured by a psychological standardized scale in this study, appeared to be a significant negative factor for resumption of sexual activity. Although drugs may affect sexuality, their effect in this study could not be evaluated well, due to variation of symptoms and psychosocial factors that also had an impact, and also lack of significant changes of drug usage before and after operation.

Eleven out of the 56 patients with preoperative sexual dysfunction resumed normal sexual activity after CABG. A sense of well-being played a role in them. In the small series of the study byAlthof*et al*, three (33.3%) of the nine previously sexually inactive patients resumed sexual activity between four and 12 months after surgery (11).

The percentage of CABG patients in our study who resumed sexual activity was lower than the alleged 73-79 percent among patients who suffered from myocardial infarction reported in previous studies (17). Apparently, all of the 19 patients in the series of Johnston *et al *(18) resumed sexual activities. In this series, among those who resumed sexual activity, 56% decreased the frequency, which is higher than the 44% to 54% observed among myocardial infarction patients (17). The average time of resumption of sexual activity was 8 weeks, less than the time among myocardial infarction patients (10.8 weeks). Johnston *et al *(18) reported similar experience (5.7 v.s. 9.4 weeks). The higher percentage of patients who returned to sexual activity and shorter time of resumption after CABG may be due to fewer symptoms, more activity recommended, preoperative expectation of improvement of physical ability, and possibly less fear. Fear as one of the reasons for lack of resumption of coitus by some of the CABG patients was not statistically significant; other factors may predominate in the decision to resume sexual activity. The incidence of fear among patients who received and those who did not receive sexual instructions was similar (18% vs. 17%). Possibly the instructions were not comprehensive enough to alleviate all worries and anxieties.

About a quarter of the patients expressed sexual concerns while hospitalized. Still, for many patients during hospitalization, the primary concerns may be those of survival and return to home. The hierarchy of needs is self-preservation first, then the needs of love and belonging. This should not be a reason for omitting the initial sexual counseling before discharge from the hospital, but indicates the need for follow up, especially for those who may not express sexual concerns early. 

Thurer stated that the sexual adjustment of 10 patients did not improve after coronary bypass surgery as measured by the structured and scaled interview for measurement of maladjustment. Sexual counseling was suggested. (19).

In our study, the CABG operation and on-pump procedure had the most potent effect in decreasing the sexual activity of patients among different methods of surgery. Similarly, Mohamed et al examined 100 patients with CABG, and found that off-pump procedure resulted in better sexual performance compared with on-pump method (20). 

Several patients in our study who received sexual instructions prior to discharge from the hospital were not sexually active preoperatively. Though such counseling may be pertinent for certain patients whose exercise tolerance may have improved postoperatively, it should be based on a preoperative sexual history complementing the overall medical history. Papadopoulos stated that less than one-quarter of the patients reported cardiac symptoms during coitus, while half of the post-myocardial infarction patients did the same (18). This is an expected result of the revascularization. In our series, 31% had angina during intercourse, while Johnston *et al*.(18) reported an incidence of 21%. 

Jenkins *et al *(2) reported that after surgery 81% of patients stated they were receiving as much affection as they would like from the people with whom they lived. Report of increased sexual satisfaction was positively correlated with the level of happiness in marriage. Furthermore, postoperative resumption of sexual activity was related to improvement of the emotional relationship of the couples and return to work of patients employed preoperatively. Comprehensive sexual counseling is still not being addressed adequately. The role and responsibility of the physician and the rehabilitation nurse became evident, together with the need for the patients partner to participate in counseling. 

Finally, it should always be kept in mind that erectile dysfunction may be early marker of atherosclerotic cardiovascular disease, and must prompt every physician to evaluate the patient for coronary artery disease (21).

**Suggestion**

According to high prevalence of sexual dysfunction, we suggest additional studies to evaluate the role of sex hormones. Also, a study using a drug intervention for prevention and treatment of sexual dysfunctions is suggested.
